# A model for predicting physical function upon discharge of hospitalized older adults in Taiwan—a machine learning approach based on both electronic health records and comprehensive geriatric assessment

**DOI:** 10.3389/fmed.2023.1160013

**Published:** 2023-07-21

**Authors:** Wei-Min Chu, Yu-Tse Tsan, Pei-Yu Chen, Chia-Yu Chen, Man-Ling Hao, Wei-Chan Chan, Hong-Ming Chen, Pi-Shan Hsu, Shih-Yi Lin, Chao-Tung Yang

**Affiliations:** ^1^Department of Family Medicine, Taichung Veterans General Hospital, Taichung, Taiwan; ^2^Education and Innovation Center for Geriatrics and Gerontology, National Center for Geriatrics and Gerontology, Ōbu, Japan; ^3^School of Medicine, National Yang Ming Chiao Tung University, Taipei, Taiwan; ^4^Department of Post-Baccalaureate Medicine, College of Medicine, National Chung Hsing University, Taichung, Taiwan; ^5^Geriatrics and Gerontology Research Center, College of Medicine, National Chung Hsing University, Taichung, Taiwan; ^6^Department of Occupational Medicine, Taichung Veterans General Hospital, Taichung, Taiwan; ^7^Department of Computer Science, Tunghai University, Taichung, Taiwan; ^8^Department of Applied Mathematics, Tunghai University, Taichung, Taiwan; ^9^Center for Geriatrics and Gerontology, Taichung Veterans General Hospital, Taichung, Taiwan; ^10^Research Center for Smart Sustainable Circular Economy, Tunghai University, Taichung, Taiwan

**Keywords:** physical function, machine learning, older adults, prediction model, comprehensive geriatric assessment

## Abstract

**Background:**

Predicting physical function upon discharge among hospitalized older adults is important. This study has aimed to develop a prediction model of physical function upon discharge through use of a machine learning algorithm using electronic health records (EHRs) and comprehensive geriatrics assessments (CGAs) among hospitalized older adults in Taiwan.

**Methods:**

Data was retrieved from the clinical database of a tertiary medical center in central Taiwan. Older adults admitted to the acute geriatric unit during the period from January 2012 to December 2018 were included for analysis, while those with missing data were excluded. From data of the EHRs and CGAs, a total of 52 clinical features were input for model building. We used 3 different machine learning algorithms, XGBoost, random forest and logistic regression.

**Results:**

In total, 1,755 older adults were included in final analysis, with a mean age of 80.68 years. For linear models on physical function upon discharge, the accuracy of prediction was 87% for XGBoost, 85% for random forest, and 32% for logistic regression. For classification models on physical function upon discharge, the accuracy for random forest, logistic regression and XGBoost were 94, 92 and 92%, respectively. The auROC reached 98% for XGBoost and random forest, while logistic regression had an auROC of 97%. The top 3 features of importance were activity of daily living (ADL) at baseline, ADL during admission, and mini nutritional status (MNA) during admission.

**Conclusion:**

The results showed that physical function upon discharge among hospitalized older adults can be predicted accurately during admission through use of a machine learning model with data taken from EHRs and CGAs.

## 1. Introduction

The world’s population is rapidly aging, particularly in developed countries (1). Taiwan is one of the developed countries which has witnessed the most rapid rise in the speed of the aging process (2). To improve the quality of life in older adults, the concept of healthy aging has become a global trend (3). Accompanied with aging, disability in later life becomes a roadblock towards the pursuiy of healthy aging. According to previous literature, disability is associated with less frequent social engagement (4), more depressive symptoms (5), multiple co-morbidities and even death (6).

Additionally, older adults are hospitalized more easily. Nowossadeck found that the aging of the population increased the number of hospitalizations for all of the diagnoses studied (7). Yet even hospitalization itself has become one of the risk factors which could lead to disability, particularly for older adults experiencing frailty (8). The mechanisms surrounding hospitalization due to disability could be older age (9), the severity of acute illness, geriatric conditions, cognitive impairment and delirium (10–12).

With advancing technology and improved medical informatics, some researchers have predicted adverse outcomes in hospitalized patients based upon electronic health records (EHRs), however data pulled from EHRs also have some limitations (13, 14). Therefore, many scientists now use a machine learning model to predict adverse outcomes in older adults (15, 16).

In recent years, multiple machine learning (ML) models have been developed to help predict physical function in older adults. Lin et al. (17) in Taiwan discovered that an ML-based method provides a promising and practical computer-assisted decision-making tool for predicting ADL amongst 313 patients admitted to the post-acute care (PAC) unit due to stroke. Kim et al. (18) in Korea also found that ML algorithms, particularly deep neural networks (DNN), can be useful for predicting the motor outcomes amongst 1,056 stroke patients in the upper and lower limbs at 6 months. Additionally, Cao et al. (19) in China used an ML-based measure of biological aging (BA) for middle-aged and older Chinese adults, with this ML-BA model being significantly associated with disability during the basic activities surrounding daily living, instrumental activities of daily living, lower extremity mobility and upper extremity mobility, as well as mortality.

However, for the ML models mentioned when predicting physical function among older adults, most were developed for community dwelling older adults, or stroke patients in a PAC unit. There is no current ML model predicting physical function during discharge among hospitalized older adults. Thus, the objectives of this study were: (1) to select appropriate features predicting physical function upon discharge of hospitalized older adults; and (2) to build up a prediction model through different ML algorithms, and then subsequently choose the most appropriate one. Thus, we aimed to build a physical function upon discharge prediction model for the hospitalized older adults based on machine learning, using a combination of EHRs and comprehensive geriatric assessments (CGAs).

## 2. Methods

### 2.1. Dataset

Our research dataset was provided by the Clinical Data Center of Taichung Veterans General Hospital. We enrolled all older adults who were admitted to our geriatric care unit during the period from January 1, 2012 to December 31, 2018. During hospitalization we collected all patient data regarding general demographics, medical history, blood examination, medication information and CGAs. Multiple assessments were performed in CGA for older adults, including physical evaluation, psychological evaluation, functional evaluation and social evaluation. The parameters of CGA included age, gender, body mass index (kg/m^2^), education level, marital status, caregiving support and measurement data. The measurement data involved cognitive impairment (defined by scores <24 on the Chinese version of the mini-mental state examination, MMSE), mood disorder (defined by scores ≥2 on the 5-item Chinese geriatric depression scale, GDS-5), medical condition (defined by the Charlson comorbidity index, CCI), polypharmacy (defined as currently using >4 drugs), malnutrition (defined by scores <12 on the mini-nutritional assessment-short form, MNA-SF), physical function (assessed by the Barthel index of activities of daily living, ADL and the Lawton instrumental activities of daily living scale, IADL), as well as frailty in accordance with cardiovascular health study (CHS) definition of the frailty phenotype, which was evaluated based upon the presence of three or more of the following criteria: weight loss, low physical activity, exhaustion, weakness (hand grip strength), and slowness (walking speed). In order to avoid redundant data collection from the same person, for those having multiple hospitalization data, only data from the latest hospitalization were retrieved. Participants with missing data were excluded. The final dataset contained a total of 1,755 patients with non-redundant data. We used collected EHR and CGA data during first 2 days upon admission and developed a prediction model of physical function during 2 days before discharge among each hospitalized participant. The study was conducted according to the guidelines of the Declaration of Helsinki, and approved by the Institutional Review Board (or Ethics Committee) of Taichung Veterans General Hospital (protocol code TCVGH-IRB CE20234A, date of approval: August 13, 2020).

### 2.2. Data pre-processing

The initial data were basic information, date of hospitalization and discharge, medical history, data files of various test values. We used the pandas package of python to convert the hospitalization and discharge dates, remove non-training features, and used the matplotlib package to visualize the data for subsequent data exploration. Through data observation, it is known that the proportion of missing values of some data features is extremely high. After the expert meeting, it was decided to remove them. Due to the characteristics of machine learning, filling in the value that should not appear in one feature can make the classifier learn that the value is a missing value, so we filled with “−999” for the remaining missing values.

### 2.3. Machine learning and prediction model development

A total of 52 potential factors were used to predict the probability of physical disability upon discharge of the elderly. An expert group consisting of geriatrician, clinical physician, professor in informatics and data analyst was gathered before the study. We had regular meeting with members of the expert group, each feature was viewed and discussed by all members and selected from previous experience and research. We used 3 different models to predict physical function upon discharge among the older adults. These models included algorithms of random forest, XGBoost and logistic regression.

### 2.4. Random forest

Random forest models are a combination of tree predictors in which each tree depends on the values of a random vector sampled independently and having the same distribution for all trees in the forest (20). The concept of random forest is to construct multiple decision trees and weaken their classification ability by combining many weak classifiers into a strong classifier, which is a strong classifier whose sample classification accuracy is above α ( α∈[0,1] ), and on the contrary, when it is below α , we call it a weak classifier, α is usually around 0.8, and this approach is also called integrated learning. The generalization error for forest models converges to a limit as the number of trees in the forest becomes larger. The generalization error of a forest model of tree classifiers depends on the strength of the individual trees in the forest and the correlation between them. Using a random selection of features to split each node yields error rates that compare favorably to Adaboost, but are more robust with respect to noise. The data set X of dimension m×k is sampled by bagging L training sets $X_{1},\ldots ,X_{L}$ , and each self-sampling X has about 36.8% of the data not sampled to $X_{i}$ , i=1,…,L is called out-of-bag data. $\;X_{1},\ldots ,X_{L}$ are all trained with the CART algorithm, with some restrictions to weaken the decision tree capability. In the end, each decision tree has its own predicted answer, and the answer with the largest proportion is chosen as the final predicted answer, in a way called voting majority.

### 2.5. XGBoost

The full name of is extreme gradient boosting (extreme gradient boosting). The eXtreme gradient boosting (XGboost) algorithm is also an algorithm that extends a decision tree, constructing multiple weak decision trees into a strong classifier, which is also known as integrated learning (21). Tree boosting is a highly effective and widely used machine learning method. Unlike random forest model, which is a bagging method applied in the random forest section, where multiple training sets are extracted by self-sampling and trained into independent classifiers, XGBoost is a weak decision tree classifier in the first step, and then develops the classifier in the second step by using the error of the classifier in the first step, with the goal of reducing the error of the classifier in the previous step, and then a strong classifier by analogy. This approach is called boosting. We define the data set as X , set L classifiers (training set), $e_{i}$ , i=1,…,L−1 as the residuals of the classifiers, and assume that the data set is $X={\left(x_{1},x_{2},\ldots ,x_{n}\right)}^{T}$ , corresponding to the actual value of $Y={\left(y_{1},y_{2},\ldots ,y_{n}\right)}^{T}$ . For $x_{i}$ , the predicted output of the model is written as a function as follows:


(1) $$f_{t}\left(x_{i}\right)={\hat{y}}_{i}{}^{\left(t\right)}$$

where i=1,…,n , and t denotes the model at the first step and defines $f_{0}\left(x_{i}\right)=0$ .

Therefore, the total output of $x_{i}$ through each step of the model can be written as the following equation:


(2) $${\hat{y}}_{i}{}^{\left(t\right)}={\hat{y}}_{i}{}^{\left(t-1\right)}+f_{t}\left(x_{i}\right)$$

where i=1,…,n . The object function of XGBoost is defined as the loss function, and the regularization term $\Omega \left(f_{t}\right)$ , which is used to control the model to avoid overfitting, can be expressed as the following equation:


(3) $$J^{\left(t\right)}=\overset{n}{\underset{i=1}{\sum }}L\left(y_{i},{\hat{y}}_{i}{}^{\left(t\right)}\right)+\Omega \left(f_{t}\right)$$

The loss function $e_{i}$ can be used in many ways, such as mean squared error (MSE). And the regularization term is the following equation:


(4) $$\Omega \left(f_{t}\right)=\gamma T_{t}+\frac{1}{2}\lambda \overset{T_{t}}{\underset{j=1}{\sum }}w_{j}^{2}$$

The equation can set the parameters $\gamma ,\lambda ,T_{t}$ is the total number of model leaf nodes at step t , labeled 1,…,T , $w_{j}$ is the weight of leaf node number j , which is also the value of model leaf node output, $j=1,\ldots ,T_{t}$ . Then the loss function L is expanded to the second order by Taylor expansion, so the target function can be written as Eq. (5)


(5) $$J^{\left(t\right)}\approx \overset{n}{\underset{i=1}{\sum }}L\left(y_{i},{\hat{y}}_{i}{}^{\left(t\right)}\right)+g_{i}f_{t}\left(x_{i}\right)+\frac{1}{2}h_{i}^{2}{\left(f_{t}\left(x_{i}\right)\right)}^{2}+\Omega \left(f_{t}\right)$$

where $g_{i}=\frac{\partial L\left(y_{i},{\hat{y}}_{i}{}^{\left(t-1\right)}\right)}{\partial {\hat{y}}_{i}{}^{\left(t-1\right)}}$ , $h_{i}=\frac{\partial^{2}L\left(y_{i},{\hat{y}}_{i}{}^{\left(t-1\right)}\right)}{\partial {\left({\hat{y}}_{i}{}^{\left(t-1\right)}\right)}^{2}}$ , which are the derivatives of $L\left(y_{i},{\hat{y}}_{i}{}^{\left(t-1\right)}\right)$ for the primary and secondary derivatives of ${\hat{y}}_{i}{}^{\left(t-1\right)}$ . The data set X may have multiple data classified to the same leaf node, and they all have the same output after input to the model, except for their $g_{i}$ and $h_{i}$ .

XGBoost describes a scalable end-to-end tree boosting system which is used widely by data scientists to achieve state-of-the-art results on many machine learning challenges. Its authors have proposed a novel sparsity-aware algorithm for sparse data and a weighted quantile sketch for approximate tree learning, while also providing insights on cache access patterns, data compression and sharding in order to build a scalable tree boosting system. By combining all these insights, XGBoost scales beyond billions of examples using far fewer resources than existing systems. In our case, we used XGBClassifier to build the model, and for the Hyperparameters setting we set the scale at_pos_weight to = 60 in order to make sure the sample be more balanced than the default setting.

### 2.6. Logistic regression

Logistic regression is a classification method that minimizes the residuals between the actual and predicted values by a least square method (22). Logistic regression is the simplest form of binary logistic regression, which follows the linear concept of linear regression. This type of statistical model (also known as logit model) is often used for classification and predictive analytics. Logistic regression estimates the probability of an event occurring, such as voted or did not vote, based on a given dataset of independent variables.

The dimension of the data set X is n×k . There are n data and k features, and the dimension is n×k . The dimension of Y is the set of n×1 categories, and Y has only two categories, 0, 1. We want to find a boundary formed by the linear combination of variables, the dependent variable is bounded between 0 and 1. Therefore, we can assume that the probability of occurrence of category 1 is p=P(Y=1|X) , and the probability of non-occurrence of category 1 is 1−p=P(Y=0|X), and the ratio p/(1−p) is statistically called The logarithm of the logistic regression is called the log-odds, also known as the “logit” function.


(6) $$\mathrm{logit}\left(p\right)=\log\frac{p}{1-p}=\beta_{0}+X\beta$$

The intercept β is usually added to β to make its dimension (k+1)×1 and a row vector of all 1’s is added to the data set X to make its dimension n×(k+1) for the convenience of subsequent calculations. Then, in order to convert these linear combinations into probabilities, they are defined by the domain (−∞,∞) and the value domain is [0,1] of the Sigmoid function.

### 2.7. Data analysis through machine learning

The integrated data were divided into training and testing sets at a 7:3 ratio, and the discharge ADL value >50 was defined as the classification basis for the binary classifier. We used sklearn kit in python for model testing in logistic regression and random forest, and we used kit in python for model testing in XGBoost. At the same time, a regression model was set up for purposes of accurate ADL prediction. After the classification model had been set up, the confusion matrix and various indicators for model evaluation were used, with the regression model using the residual distribution map and various indicators for model evaluation. Also, because ADL does not seem to change a lot in a short period, we performed sensitivity analysis which excluded ADL upon admission as a feature by using random forest.

## 3. Results

**Figure 1:**
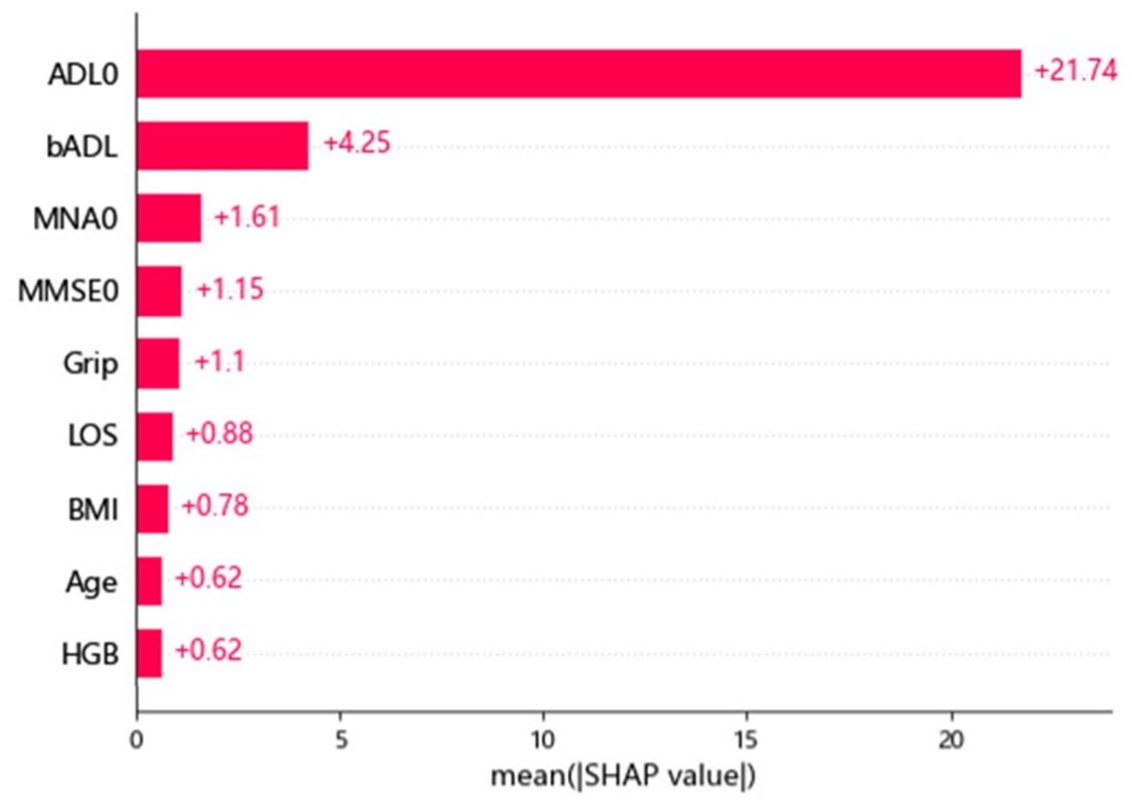
Features of importance for building up the regression model by XGBoost. ADL, activity of daily living; BMI, body mass index; LOS, length of stay; MMSE, mini mental status examination; MNA, mini nutritional assessment; HgB, hemoglobin.

Table 1 shows the demographic and clinical characteristics of the 1,755 older adults, including 702 participants with an ADL ≤50 upon discharge and 1,053 participants with an ADL >50 upon discharge. Their mean age was 80.68 years, with a male predominance (62.3%). Table 2 shows the difference in accuracy, cv accuracy, MSE and RMSE for prediction of accurate physical function upon discharge among all 3 models, XGBoost, random forest and logistic regression. The accuracy of prediction was 87% for XGBoost, 85% for random forest and 32% for Logistic regression. Figure 1 shows the features of importance for building up the regression model by XGBoost. ADL upon admission, baseline ADL and MNA upon admission were the top 3 features of importance.

**Table 1:** Demographic characteristics of participants.

Patient characteristics	Total (*n* = 1,755)	ADL when discharge ≤50 (*n* = 702)	ADL when discharge >50 (*n* = 1,053)
	*n* (%)/mean (sd)	*n* (%)/mean (sd)	*n* (%)/mean (sd)
Male	1,094 (62.3%)	425 (60.5%)	669 (63.5%)
Age	80.68 (±8.07)	83.34 (±7.61)	78.91 (±7.89)
BMI	23.87 (±4.27)	22.75 (±4.37)	24.49 (±4.08)
Creatinine	1.47 (±1.55)	1.47 (±1.41)	1.46 (±1.65)
EGFR	70.41 (±35.40)	71.98 (±41.60)	69.39 (±30.71)
Sodium	138.17 (±5.95)	137.49 (±6.87)	138.66 (±5.13)
GPT	34.97 (±66.67)	31.94 (±48.56)	37.14 (±77.01)
Grip strength	17.40 (±7.83)	12.66 (±5.91)	19.46 (±7.67)
Baseline ADL	73.01 (±32.27)	44.88 (±32.54)	91.77 (±12.27)
ADL when admission	48.25 (±32.29)	15.72 (±15.35)	69.94 (±20.11)
MMSE when admission	20.33 (±6.71)	15.06 (±6.23)	22.80 (±5.37)
MNA when admission	20.56 (±5.09)	16.59 (±4.33)	23.20 (±3.62)
Length of hospitalization	11.60 (±9.06)	14.25 (±10.19)	9.84 (±7.73)
CHS score	2.52 (±1.54)	2.89 (±1.59)	2.27 (±1.46)
Walking ability	1.13 (±1.45)	2.25 (±1.52)	0.38 (±0.74)

ADL, activity of daily living; BMI, body mass index; EGFR, estimated glomerular filtration rate; MMSE, mini mental status examination; MNA, mini nutritional assessment; CHS, cardiovascular health study.

**Table 2:** Accuracy, cv accuracy, MSE and RMSE of all prediction models for accurate prediction of physical function upon discharge.

	Accuracy	Cv accuracy (cv = 10)	MSE	RMSE
XGBoost	0.87	0.87	140.88	11.87
Random forest	0.85	0.86	142.87	12.11
Logistic regression	0.32	0.70	309.85	17.60

MSE, mean square error; RMSE, root mean square error.

**Figure 2:**
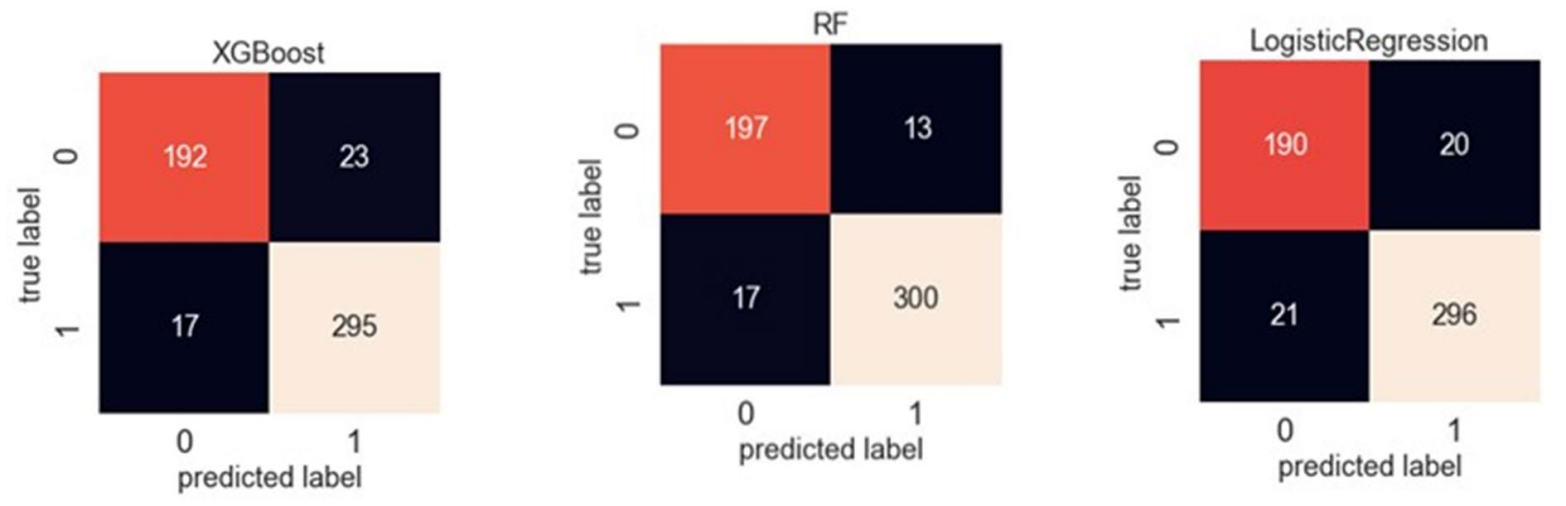
Confusion matrix of XGBoost, random forest and logistic regression in classification models.

Table 3 reveals the accuracy and macro *F*-1 score of the classification models. Accuracy for random forest, logistic regression and XGBoost were 94, 92 and 92%, respectively. Confusion matrix in different prediction models is shown in Figure 2. The result of sensitivity analysis which excluded ADL upon admission as a feature by using random forest showed that the accuracy was still high (0.89 vs. 0.94) after excluding ADL upon admission (Supplementary Table 1).

**Table 3:** Accuracy, precision, recall and *F*-1 score of XGBoost, random forest and logistic regression in classification models.

	Class 0 (ADL <= 50)			Class 1 (ADL > 50)			Accuracy	Macro fl score
	Precision	Recall	*F*1-score	Precision	Recall	*F*1-score		
Random forest	0.92	0.94	0.93	0.96	0.95	0.95	0.94	0.94
Logistic regression	0.9	0.9	0.9	0.94	0.93	0.94	0.92	0.92
XGboost	0.92	0.89	0.91	0.93	0.95	0.94	0.92	0.92

**Figure 3:**
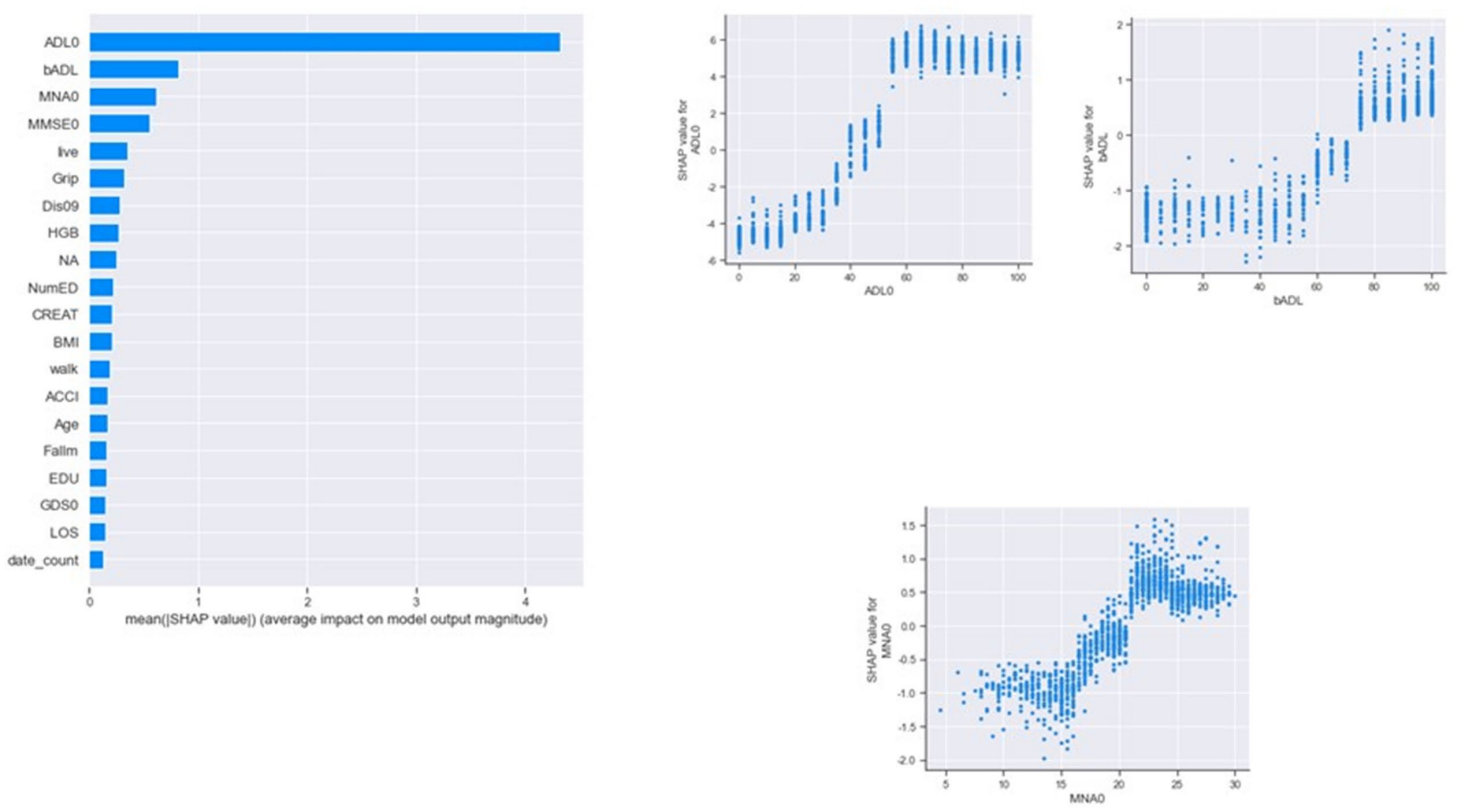
Features of importance by XGBoost in classification models. ADL, activity of daily living; BMI, body mass index; LOS, length of stay; MMSE, mini mental status examination; MNA, mini nutritional assessment; HgB, hemoglobin; NumED, number of emergency department visit; ACCI, age-adjusted Charlson comorbidity index; EDU, educational level; GDS, geriatric depression scale.

**Figure 4:**
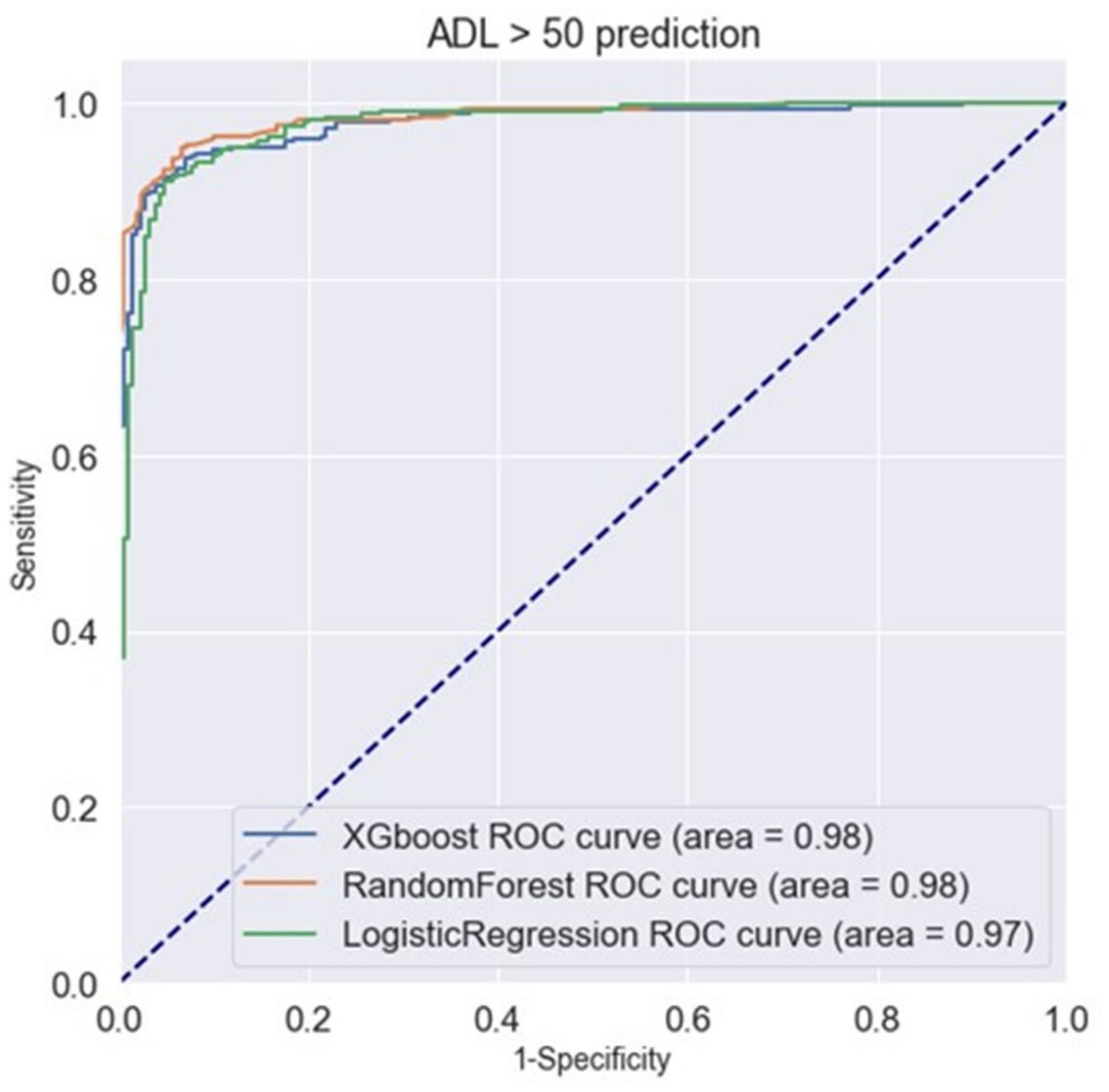
ROC curve of XGBoost, random forest and logistic regression in classification models.

Classifying through use of the algorithm, the importance of the features in the classification process is calculated. From Figure 3, we found that ADL upon admission, baseline ADL and MNA upon admission were the top 3 features of importance. Figure 4 shows the ROC curve of XGBoost, random forest and logistic regression. The XGBoost and random forest models both had an auROC of 98%, while logistic regression had an auROC of 97%.

## 4. Discussion

To the best of our knowledge, this is the first study using both EHR and CGA to help predict physical function upon discharge among hospitalized older adults. The results show that when combined with the key clinical features at baseline and during admission, using the XGBoost and random forest ML models could help predict accurate physical function upon discharge. For categorical prediction, using the XGBoost, random forest and logistic regression ML models resulted in good prediction. We therefore believe that this model build can help healthcare professionals better understand in advance each patient’s physical function upon discharge, thus allowing for better discharge planning in connection with home health care services.

The results of our model seem to be better than previous models which have been built and discussed in the available literature. Lin et al. (17) used logistic regression, support vector machine and random forest models to predict ADL upon discharge based on 15 rehabilitation assessments among post-stroke patients in the PAC unit of a Taiwan hospital, with the results showing the area under curve (AUC) of LR, RF and SVM to be 0.79, 0.79 and 0.77, respectively. In Korea, Kim et al. (18) used 14 input variables to predict upper limb function and lower limb function among post-stroke patients using DNN, logistic regression and random forest. They came up with results showing DNN to be the best prediction model, with an AUC of 0.874 for upper limb function and 0.822 for lower limb function (18). In Italy, Verrusio et al. (23) used a combination of the two SVMs to predict functional outcome a year later among community-dwelling older adults undergoing rehabilitation, and reached an accuracy level of 84%, when compared to the results of 67% seen in linear regression models. Thus, from our results, the ML algorithm cannot only predict relatively long-term outcomes, but can also predict short-term outcomes as well, which is more valuable for healthcare professionals in acute care settings.

This is the first study ever performed using CGAs and EHRs together with machine learning to help predict physical function upon discharge among hospitalized older adults. CGA is a multi-dimensional, multi-disciplinary diagnostic and therapeutic process conducted to determine the medical, psychological and functional problems of older people with frailty so that a coordinated and integrated plan for treatment and follow-up can be developed (24). Currently, CGA is used widely and regarded as the gold standard in the care of frail, older patients in hospitals (25). CGA has also been used to identify any risk of adverse events, such as mortality, functional decline, surgical complications and chemotherapy toxicity among cancer patients (26). Using CGA in machine learning to help predict outcomes among older adults has been put into practice more widely in recent years. Schiltz et al. (27) discovered that IADL limitation could be used in a random forest model to predict 30 days readmission among hospitalized older adults. Even more so, Sena et al. (28) in Brazil found that CGA could be used to build up a simplified predictive model aimed at estimating the risk of early death in older cancer patients. Iwamoto et al. (29) used machine learning-based clinical prediction rules for the identification of ADL dependence in stroke patients under rehabilitation, resulting in moderate predictive ability. CGA has also been used in machine learning to better evaluate older patients with atrial fibrillation (30). Our previous work has also showed that CGA combined with EHR can predict fall risk among the older adults (16). Future studies are still warranted for both identification and intervention in the promotion of physical function during hospitalization after any machine learning prediction.

Along with baseline ADL and ADL upon admission, we found that one’s nutritional status upon admission was a quite important feature in both lineal and classification models. Nutritional status is a known factor for the maintenance of functional status, with malnutrition being a risk factor for further sarcopenia (31), frailty (32), disability (33) and mortality (34). Obesity also remains a risk as well. Recently, a study conducted in Brazil and the United Kingdom discovered that an elevated body mass index (BMI) and increased waist circumference increased the odds of disability in both populations (35). Our findings regarding malnutrition should remind healthcare professionals to pay more attention to nutritional status upon admission among hospitalized older adults, due to the fact that it is highly associated with further functional outcomes upon discharge.

### 4.1. Strength and limitations

Our study has some limitations. First, the investigation was limited to data from a single hospital, thus external validity should be interpreted with caution. Further testing our models using data from other hospitals in other regions is needed in order to establish external validity. Secondly, certain important factors related to physical function were not considered, such as the caregiver-related factor. Therefore, any future projects should include both these important factors in order to reach a better physical function prediction. Third, the generalizability of this method is questionable because most healthcare professionals may not use CGA as a routine tool of assessment for older patients. However, more and more CGA are being used in clinical settings, even in clinical trials (36). Thus, we believe that our model will be useful for prediction of physical function upon discharge among older adults in the near future.

### 4.2. Implications

The results of our study show that the prediction of physical function upon discharge, when performed during admission, is possible through use of a machine learning model. For clinical healthcare professionals caring for older adults, we believe our prediction model could help with shared decision making, particularly for discharge planning performed in advance. Additionally, predictive physical function could be regarded not only as a potential goal of recovery, but also for examining the clinical process and quality of care through continuous monitoring.

There was new model developed through our research, and we did not manage adaptations of the developed model because the results were quite convincing after initial model building. We will keep managing adaptations of the developed model in future study and further we would like to build our own model for prediction.

## 5. Conclusion

We were able to predict physical function upon discharge among hospitalized older adults through a combination of EHRs and CGAs. We found that ADL upon admission, ADL at baseline and MNA upon admission are the 3 important factors involved in the prediction model. The accuracy of the XGBoost and random forest model evaluations reached 87% and 85%, respectively, based upon 52 features.

In any future adjustments of the model, there should be several directions taken. First, we would like to add more features to the model, such as diagnosis of chronic disease and medication use, to improve even more the accuracy of the model prediction. Secondly, we would seek to explore the application of feature selection in different machine learning models among the older adults, because from our results, it was shown that feature selection was complicated as well as important. Third, we will perform any validations in different settings, including acute wards, chronic wards and intensive care units in order to better test our models.

## Data availability statement

The data analyzed in this study is subject to the following licenses/restrictions: the datasets used and analyzed during the current study are not publicity available, but are available from the corresponding author on reasonable request with the permission of Taichung Veterans General Hospital, Taiwan. Requests to access these datasets should be directed to S-YL, sylin@vghtc.gov.tw.

## Ethics statement

The studies involving human participants were reviewed and approved by Institutional Review Board (or Ethics Committee) of Taichung Veterans General Hospital. Written informed consent for participation was not required for this study in accordance with the national legislation and the institutional requirements.

## Author contributions

W-MC, S-YL, and C-TY conceived of the study and supervised all aspects of its implementation. W-MC and P-YC completed the analyses and drafted the content. Y-TT, H-MC, and P-SH assisted with the study design and revised the content. C-YC, M-LH, and W-CC assisted with statistical analysis and revised the content. W-MC, Y-TT, P-YC, C-YC, M-LH, W-CC, H-MC, P-SH, S-YL, and C-TY helped to conceptualize ideas, interpret findings and review drafts of the manuscript. All authors contributed to the article and approved the submitted version.

## Funding

This work was supported by Taichung Veterans General Hospital, Taiwan (Grant number: TCVGH-T1117803 and TCVGH-T1127809 awarded to W-MC). The funders had no role in the design of the study; in the collection, analyses or interpretation of data; in the writing of the manuscript; or in the decision to publish the results.

## Conflict of interest

The authors declare that the research was conducted in the absence of any commercial or financial relationships that could be construed as a potential conflict of interest.

## Publisher’s note

All claims expressed in this article are solely those of the authors and do not necessarily represent those of their affiliated organizations, or those of the publisher, the editors and the reviewers. Any product that may be evaluated in this article, or claim that may be made by its manufacturer, is not guaranteed or endorsed by the publisher.
