# Supplementary material for: A model for predicting physical function upon discharge of hospitalized older adults in Taiwan—a machine learning approach based on both electronic health records and comprehensive geriatric assessment
Source: Front Med (Lausanne). 2023 Jul 21;10:1160013. doi: 10.3389/fmed.2023.1160013 (PMC10400801; doi:10.3389/fmed.2023.1160013)
Supplement: Supplementary file 1 [file Table_1.DOCX]

Supplementary Table

Supplementary Table 1. Sensitivity analysis with model development without ADL upon admission by Random forest

|  | Initial features | | | |  | Features without ADL upon admission | | | |
| --- | --- | --- | --- | --- | --- | --- | --- | --- | --- |
|  | precision | recall | f1-score | support |  | precision | recall | f1-score | support |
| ADL <= 50 | 0.96 | 0.9 | 0.93 | 215 |  | 0.89 | 0.84 | 0.87 | 215 |
| ADL > 50 | 0.93 | 0.98 | 0.95 | 312 |  | 0.9 | 0.93 | 0.91 | 312 |
|  |  |  |  |  |  |  |  |  |  |
| accuracy |  |  | 0.94 | 527 |  |  |  | 0.89 | 527 |
| macro avg | 0.95 | 0.94 | 0.94 | 527 |  | 0.89 | 0.89 | 0.89 | 527 |
| weighted avg | 0.95 | 0.94 | 0.94 | 527 |  | 0.89 | 0.89 | 0.89 | 527 |
